# Leg Power As an Indicator of Risk of Injury or Illness in Police Recruits

**DOI:** 10.3390/ijerph13020237

**Published:** 2016-02-19

**Authors:** Robin Orr, Rodney Pope, Samantha Peterson, Benjamin Hinton, Michael Stierli

**Affiliations:** 1 Tactical Research Unit, Bond University, Gold Coast, QLD 4226, Australia; rpope@bond.edu.au; 2 Faculty of Health Sciences and Medicine, Bond University, Gold Coast, QLD 4226, Australia; samantha.peterson@student.bond.edu.au; 3 New South Wales Police Department, Sydney, NSW 2010, Australia; hint1ben@police.nsw.gov.au (B.H.); stie1mic@police.nsw.gov.au (M.S.)

**Keywords:** tactical, law enforcement, vertical jump, injury prevention, fitness assessment

## Abstract

Tactical trainees, like those entering the police force, are required to undergo vigorous training as part of their occupational preparation. This training has the potential to cause injuries. In addition, the physical training, communal living and pressures of tactical training are known to induce immune suppression and have the potential to increase the risk of illness. The aim of this study was to investigate the relationship between leg power, as measured by a vertical jump (VJ), and rates of reported injuries and illnesses during police recruit training. Retrospective data from recruits (*n =* 1021) undergoing basic police recruit training at an Australian Police Force College was collected. Recruits completed a VJ assessment at the commencement of their second state of training. Formally reported illness and injuries were collected 12 weeks later, following completion of training. Correlations between VJ height and rates of reported illness and injury were low (*r =* −0.16 and −0.09, respectively) but significant (*p <* 0.005), with VJ height accounting for 2.6% and 0.8% of the variance in illness and injury rates, respectively. In terms of relative risks, recruits with the lowest recorded VJ heights were more than three times as likely as those with highest VJ heights to suffer injury and/or illness. Police recruits with lower VJ height are at a significantly greater risk of suffering an injury or illness during police basic recruit training.

## 1. Introduction

“Emergency preparedness” is not simply the ability to complete a required task, but also the capacity to do so repeatedly without experiencing excessive stress [1]. Professionals within the tactical fields rely on their physical fitness for occupational success, and competency in these physical fitness components must be demonstrated to be accepted into their field [2]. As such training for new tactical recruits must be sufficient to prepare them for their new occupation but at the same time the new recruits must be resilient enough to complete the training. Considering this, new recruits are traditionally drawn from the general population and have widely varying fitness levels. Once training commences these new recruits are subjected to a high amount of physical stress and this potentially dramatic increase in physical stress may lead to injury [3]. As such, new trainees are known to be at a higher risk of injury when compared to their trained counterparts [4,5,6,7], with these injuries typically affecting the lower limbs [8,9] and often caused by increase in physical conditioning requirements, the complexity of new physical tasks, reduced opportunity for recovery and resulting increased risk of overtraining [4,5,6,7]. 

Exercise also has a direct effect on the immune system, however these effects can vary and are influenced by several factors, such as genetics, environment and even exercise intensity [10,11]. While moderate exercise has been suggested to provide a protective effect, repeated bouts of intense exercise has been suggested to result in immune dysfunction. While, there are still few studies that have been able to show a direct link between exercise-induced immune depression and increased incidence of illness in athletes, research has confirmed that a single, acute bout of prolonged, strenuous exercise has a temporary depressive effect on immune function [10]. This has been associated with an increased incidence of infection in the weeks following an event [10]. In addition, following periods of chronic training at high workloads, aspects of the immune system have been found to be depressed and while not clinically immune deficient, the result may lead to an increased risk of more mundane illnesses (like influenza) [10]. As such, less fit trainees, who would have to work at a higher intensity to complete a given task, may be at greater risk of sustaining an illness. 

All tactical populations have strenuous physical tasks they must perform. For police officers in particular, several important tasks involve leg power, such as jumping and negotiating stairs to chasing fleeing suspects in the streets [2]. The current literature shows that certain aspects of physical fitness test scores are predictors of job performance [2,12,13,14], with some research finding that jumping ability, specifically, is a good predictor of physical fitness [1,15,16]. Considering this, several variations of the Vertical Jump (VJ) test have been shown to be valid and reliable measures of leg power [15], associated with job performance in a range of contexts [12,13,14,17] and potentially prognostic for injury recovery [18]. 

Given that recruits: (1) may be at a higher risk of injury if they have lower levels of fitness due to increases in physical conditioning requirements; (2) may have potential reductions in immune function due to exercise; and (3) have a leg power requirement to complete police tasks, and given that the VJ is considered an valid and reliable measure of leg power and occupational performance; the question arrises as to whether leg power as measured by a VJ is associated with injuries and illness sustained by police recruits during training. On this basis, the objective of this research was to investigate the correlations between leg power, measured via the VJ test, and rates of reported injuries and illnesses during police recruit training in order to establish its validity for use as a part of current police recruit fitness screening protocols.

## 2. Materials and Methods

The study used a retrospective cohort study design involving data previously collected by an Australian police college. The retrospective data consisted of vertical jump results preiosouly collected, and now stored, by the police college Physical Training Instructors and injury/illness data recorded on an electronic database. Data were collated and aligned by the police researchers (BH, MS) and made non-identifiable prior to analysis. In total, data were provided for 1041 police recruits covering the period January 2013 to June 2014. These recruits were undergoing fulltime training (12 weeks), at an Australia Police college. Trianing at the college is divided into two 12-week sessions; Sesson 1 and Session 2. Session 1 and Session 2 have similar physical training requirements (see below) but where Session 1 is predominantly theoretical and classroom-based, Session 2 has a greater physical performance requirement (defensive tactics, use of force, batton striking and marksmanship, for example). As such, Session 2 was selected as the intervention measure for this program of research. The aforementioned physical training program for Session 2 included a resistance training component (push-ups, lying pull-ups and sit-ups) and a running component consisting of long-slow distance running and long (400 m) or short (20 m) interval running (~60 min in length). Physical training sessions typically took place first thing in the morning at around 0800 h, for a one hour period, although this was subject to change if competing priorities (like range bookings) were presented and were conducted once per week. For the resistance training component the number of repetitions increased each week for five weeks followed by a progressive increase in load for five weeks. For the running component, distance increased progressively for a period of 5 weeks following which the number of repetitions for the interval sessions increased for the next 5 week period. For security reasons, no demographic information on these recruits was available, however all recruits did meet the necessary entry requirements for age (a minimum of 18 years and 4 months), had completed a health clearance from a General Practitioner and had a full medical assessment completed by an external provider. Inclusion criteria were: (a) the recruit was attending “Session 2” police recruit training; (b) the recruit had not attempted Session 2 training previously and (c) the recruit was able to complete the fitness assessments and all occupational measures. The exclusion criterion for this study was a recruit who was suffering an injury or illness at the time of initial fitness assessment, precluding a valid VJ height measurement. Ethics approval for the study was provided by the Bond University Human Research Ethics Committee (RO 1898).

### 2.1. Outcome Measures

VJ height and formally reported illness and injury status of participants constituted the primary outcome measures. The VJ is a commonly selected measure of performance and has been used to investigate lower-body explosive power, due to the ease and reliability of the assessment [19]. The VJ assessment was performed by all recruits at the commencement of Session 2 training. 

Recruits were required to reach up and touch a premarked and premounted board on the gymnasium wall at maximal vertical arm reach to determine the standing upward reach height of each recruit and normalise jump height based on height–as described by by Harman and Garhammer [20]. Recruits were instructed to perform a rapid counter movement jump with an arm swing. All recruits were instructed to perform a maximal jump and touch a premarked and premounted board on the gymnasium wall. The better of two attempts was taken and maximal jump height was recorded to the nearest 1 cm. Police Physical Training Instructors conducted all of the VJ assessments and were unaware of the research. The protocol is that described by Harman and Garhammer [20] with the exception being the best of two trials rather than three due to time constraints. 

Data pertaining to recruit injuries and illness were routinely captured through standard institutional accident and incident forms, in accordance with normal police processes, and were entered into a police electronic database. Injury was defined as physical damage to the neuromusculo-skeletal system of the body and illness a compromise of the immune system (e.g., viral or bacterial). Determination of “injury” or “illness” was made by the treating medical staff. Upon cohort graduation, the reported injuries or illnesses data were provided by police staff to the research team. 

### 2.2. Data Analysis

Upon receipt, data were first manually cleaned. Participants’ data were then divided into groups based on three outcomes: (1) participants who reported or did not report injury; (2) participants who reported or did not report illness and (3) participants who did or did not report both injury and/or illness. Raw VJ scores were used to further categorize participants in brackets of 5 cm prior to analysis. For each bracket, in each group, the numbers of trainees who did and did not report injury and illness were ascertained. The rates of injury and illness were then calculated as the percentage of trainees who reported injury or illness, respectively, during the 12-week period of the training course, and 95% confidence intervals were calculated for each of these percentage values. Where cell counts were less than three in any VJ height bracket, the results for that bracket were pooled with the results from the preceding VJ height bracket to provide more valid estimates of proportions. 

Following descriptive analysis, independent samples *t*-tests were used to investigate differences between injury- or illness- affected groups and non-affected groups in mean VJ heights. Spearman’s Rho correlations were used to determine the relationships between VJ heights and rates of injury and illness. Statistical analyses were conducted using the Statistical Package for the Social Sciences (SPSS) software v.22 (IBM Corporation, Amork, NY., USA), with alpha set *a priori* at 0.05.

## 3. Results

Of the initial 1041 results, 20 were excluded based on the exclusion criterion, leaving 1021 data sets for analysis. Injuries alone were reported by 15% (*n =* 158) of the participants, illness alone by 29% (*n =* 296) of participants, and either an injury, an illness or both by 38 % (*n =* 390) of participants. In total, 62% of participants did not report either an injury or illness. The VJ score comparisons among the various participant groups are displayed in Table 1, Table 2 and Table 3. Significantly lower mean VJ height scores were observed in those injured and those who suffered illness when compared to those not injured and who did not suffer illness (Table 4). In a clinical application, when viewed in terms of relative risks, recruits with the lowest recorded VJ heights were more than three times as likely as those with highest VJ heights to suffer injury and/or illness (Table 3). Similar trends were observed for injuries alone (Table 1) and illness alone (Table 2). 

Correlations between VJ height and prevalence of illness and injury were low (*r =* −0.16 and −0.09, respectively; Table 4) but significant (*p <* 0.005), with VJ height accounting for 2.6% and 0.8% of the variance in illness and injury rates, respectively.

## 4. Discussion

The aim of this study was to determine the relationship between leg power and risks of injury, illness or both in police recruits in order to establish its validity for use as a part of current police fitness screening protocols. The findings showed that participants who scored a VJ height in the lower part of the range of VJ scores were significantly more susceptible to injury or illness than those with greater VJ heights. This relationship is readily seen in Table 3, where only 17% of participants who reached a VJ height in the range of 60–70 cm experienced injury and/or illness, whereas 54% of those who reached a VJ height of 30–34 cm experienced injury and/or illness. This represents more than a three times greater risk or more for those reaching the lowest VJ heights when compared to those reaching the highest VJ heights, and similar relationships were observed for injury and for illness alone (Table 1 and Table 2). 

These relative risks are clinically important, despite the low but significant overall correlation between VJ height and these adverse outcomes (Table 4). Potential mechanisms for these heightened levels of risk in those exhibiting low VJ heights are discussed in some detail below. However, as a prelude to that discussion, it is important to note that heightened levels of risk like those observed in this study often represent cumulative risk differences, accruing steadily, day after day, across the training program. The actual increase in level of risk for the higher risk group (in this study, those exhibiting a low VJ height) within each individual physical activity event of the total training program may be very small, as reflected in the strengths of correlations observed in this study, for example. However, as the days pass and more and more physical activity events are completed, the risk level steadily builds, with each event adding another small amount of risk, until the final, accumulated effect of the heightened levels of risk across all physical training events can be ascertained at the end of the training program–in this case amounting to a threefold relative increase (or 37% absolute increase) in risk of injury and/or illness accruing in the low-VJ-height group by the end of the training program when compared to the high-VJ-height group. This phenomenon of differential risk accrual in different groups, over repeated exposures to physical activity events across a training program, is discussed in detail by Pope [21]. 

Nevertheless, some caution should be applied in the interpretation of the results of this study. The study has examined only VJ height as a predictor of injury and illness risk in trainees, and injury and illness causation is clearly multi-faceted, with many factors contributing to risk. Future research should examine the value of VJ height scores in a multifactorial approach which includes other potential predictor variables, in order to ascertain the unique predictive value VJ height scores might *add* to test batteries used for trainee screening and assessment. In addition, the fact that VJ height scores *predict* injury and illness risk does not mean that low leg power *causes* an increase in injury and illness risk. Rather, this measure of leg power might be an indicator or correlate of other factors that directly affect injury and illness risk, such as those we will now proceed to discuss.

### 4.1. Vertical Jump Height and Injury

Supporting the findings of our study, and our synopsis above of accumulated risk, several other studies [22,23,24] have found that fatigue increases the likelihood of injury. Neuromuscular fatigue can occur anywhere along the neuromuscular pathway and impairs the neural and muscular mechanisms [22,25]. Fatigue, particularly in the lower limbs, may result in changes in gait, causing unaccustomed musculoskeletal stress on specific body areas and can change the physical mechanics of dynamic activity [9,22,25]. One study found that leg power, measured via VJ, decreased following strenuous activity in the form of a load carriage march, negatively influencing job performance [17] while another identified decreases in VJ associated directly with knee extensor muscle fatigue [24]. Similar findings were observed by Byrne and Eston [26] whereby VJ performance decreased immediately following an exercise bout and remained reduced for up to 4 days. Recruits with lower levels of strength and power would have to work at a higher intensity to maintain a given intensity when compared to stronger recruits. As such, when performing the same training, recruits with lower levels of strength and power are likely to fatigue sooner in a clinical setting. Considering this, with leg power reduced by exercise, there is the potential for an injury to occur either during or for up to a short time after an activity that has a notable physical component (for example physical training session or defensive tactics training).

However, not all studies are in agreement with our findings. A study by Knapik *et al.* [9] examined the association between injuries in combat training and four measures of strength and one measure of leg power, measured via the VJ. Contrary to our findings, these researchers found no association between injury and the leg power measure, but stated that their findings indicated muscular endurance to be closely related to injuries [9]. It is possible that this difference is due to the greater endurance emphasis of military recruit training when compared to the police recruit training that was the focus of our study. In addition to urban operations, military recruits perform long endurance marches and patrols, while for police recruits urban and close-proximity tasks are their more common requirements; requirements where power and speed feature more prominently. 

### 4.2. Vertical Jump Height and Illness

Exercise intensity, amongst other variables concomitant with a multi-stressor environment (like communal living) [27], has a direct, yet varying, effect on the immune system [10,11]. Research shows that a single bout of acute prolonged strenuous activity has a temporary depressive effect on several immune cell functions [28]. Cellular changes during early recovery from exercise appear to weaken the potential immune response to pathogens and have been proposed to provide an “open window” for infection. With most cellular changes returning to normal in 3–24 h, this exercise-induced immune depression is not considered to put athletes in danger of serious illness however this period could be sufficient to increase the risk of the athlete picking up common infections [28].

In addition, chronic or regularly intensive exercise has its own distinct effects on the immune system. Leukocyte, neutrophil, and natural killer cells are sensitive to training load and reduce in response to increases in load thus leading to immune depression [10]. As an example, a study in 2005 looked at the incidence of upper respiratory tract infections during intensive military training [29]. Although this study found the soldiers to have a normal immune function at the end of their training course, the soldiers had a significantly decreased natural killer cells count in week 4 of training which led to an increased incidence of upper respiratory tract infections [29]. 

This potential for an increased risk of illness with normal immune function markers is understandable given findings that illness incidence associated with overtraining is not potentially due to immunosuppression itself but rather altered immune function [30].

While the exact mechanisms are still under investigation, the downstream effects of chronic high intensity physical activity have been associated with an increased risk of illness. On this basis, the physical training undertaken as part of the police training may have differentially led to greater intensity requirements in those with lower VJ heights when performing a given task. This higher intensity requirement may have contributed greater levels of immune depression or altered immunological responses in these recruits, and as such lead to a higher risk of illness [10,31]. 

### 4.3. Vertical Jump Height and Risk of Injury and Illness

Fifteen percent of recruits in this study reportedly suffered an injury, 29% an illness and 38% of participants both an injury and/or illness with lower VJ heights common across all three populations groups. The findings in our study differ from the few studies found investigating both injuries and illness in a military population [32,33,34]. In one study investigating both male and female trainee air force cadets undergoing basic training, found the number of injuries reported to be slightly higher than the number of illnesses for a given population (*n =* 846 injuries: *n =* 816 illnesses) [32]. Conversely, a study of female marine recruits found reported injuries to be notably higher (44%) than reported illnesses (22.2%) [33]. A higher number of injuries as opposed to illnesses were also found in an earlier study of army recruits whereby 19% reported an illness and 25.3% reported an injury [34]. These results differ from our study, yet may be explained by differences in training environments and contexts (intensity, type and volume) of physical training and physical activity contexts, all of which are known to be stressors which can impact on the risk of injury and illness [31]. For example, the paper investigating injuries and illness in female military populations found musculoskeletal injury rates to range from 37.2% to 61% and respiratory illness from 22.2% to 26.1% across three different data collection sites [33]. 

### 4.4. Limitations

A key limitation of our study was the lack of detail within the provided data. The data provided was limited to classification of injury or illness with no detail on the nature of the injuries and illnesses. This lack of data depth limited the injury and illness analysis, most notable the types, bodily sites and importantly injury or illness severity. More detailed data would be of benefit to provide a more detailed profile on the injuries and illnesses presented as well as to determine training time loss, rehabilitation/recovery costs and attrition rates and potential inter-practitioner differences in diagnosing injury or illness. In addition, the lack of additional information limited the study to examining VJ height scores alone as a predictor variable. In future research, it will be valuable to examine the value of VJ height scores in a multifactorial approach which includes other potential predictor variables, in order to ascertain the unique predictive value it might add to test batteries used for trainee screening and assessment.

## 5. Conclusions

This work has suggested a significant and clinically-important relationship may exist between leg power and risk of illness and injury in police recruits undertaking basic training. Both prior research findings and the findings of our study suggest that, in some tactical contexts, participants with poorer VJ scores are more likely to be less fit overall and to therefore suffer greater levels of fatigue, which lead in turn to an increased risk of injury, illness or a combination of both. On this basis, it appears the VJ test is a valid predictor and may be a useful tool to employ as part of a screening protocol assessing the risk of injury or illness in a police recruit population, however we do not yet know what level of *additional* predictive value it might add to test batteries used to screen and assess trainees, over and above other measures such as measures of aerobic fitness and prior injury history. Further research using a multifactorial approach is required to assess its utility in such batteries of tests. It is also possible that measures to increase VJ power may improve overall fitness and preparedness of trainees for police duties and reduce risks of injury or illness in the police population. However, this possibility and the notion that leg power or leg power training might contribute directly to injury and illness risks by a causal link also requires further research. 

## Figures and Tables

**Table 1:** Vertical jump score comparison of the injured and non-injured participants.

VJ Groups (cm)	Injured			Non-Injured		
	n	%	95% CI	N	%	95% CI
30–34	25	22	15.3%–30.4%	89	78	69.6%–84.7%
35–39	39	20	14.7%–25.7%	160	80	74.3%–85.3%
40–44	37	16	11.7%–21.2%	196	84	78.9%–88.3%
45–49	27	12	8.5%–17.1%	195	88	82.9%–91.5%
50–54	20	13	8.5%–19.0%	136	87	81.0%–91.5%
55–70 *	10	10	5.5%–18.1%	87	90	81.9%–94.5%
TOTAL	158	15	-	863	85	-

* Pooled results for the last three VJ brackets, due to small cell counts in order to provide a more valid estimate.

**Table 2:** Vertical jump score comparison for trainees diagnosed with an illness *versus* those not reporting or diagnosed with an illness.

VJ Groups (cm)	Reported Illness			No Reported Illness		
	n	%	95% CI	N	%	95% CI
30–34	51	45	35.9%–53.9%	63	55	46.1%–64.1%
35–39	70	35	28.9%–42.0%	129	65	58.0%–71.1%
40–44	68	29	23.7%–35.3%	165	71	64.7%–76.3%
45–49	51	23	17.9%–28.9%	171	77	71.1%–82.1%
50–54	35	22	16.6%–29.6%	121	78	70.4%–83.4%
55–59	18	25	16.2%–35.6%	55	75	64.4%–83.8%
60–70 *	3	12.5	3.5%–31.8%	21	87.5	68.2%–96.5%
TOTAL	296	29	-	725	71	-

* Pooled results for the last three VJ brackets, due to small cell counts in order to provide a more valid estimate.

**Table 3:** Vertical jump score comparison for trainees reporting injury and/or illness *versus* those reporting no injury or illness.

VJ Groups (cm)	Reported Injury/Illness			No Reported Injury/Illness		
	n	%	95% CI	n	%	95% CI
30–34	61	54	44.4%–62.4%	53	47	37.6%–55.6%
35–39	91	46	39.0%–52.7%	108	54	47.3%–61.0%
40–44	95	41	34.7%–47.2%	138	59	52.8%–65.3%
45–49	68	31	24.9%–37.0%	154	69	63.0%–75.1%
50–54	48	31	24.1%–38.4%	108	69	61.6%–75.9%
55–59	23	32	22.0%–42.9%	50	69	57.1%–78.0%
60–70 *	4	17	6.1%–36.5%	20	83	63.5%–93.9%
TOTAL	390	38	--	631	62	-

* Pooled results for the last three VJ brackets, due to small cell counts in order to provide a more valid estimate.

**Table 4:** Descriptive analysis, demographics and statistical results in each group.

Status	VJ Mean ± SD (Range)	*t*-Test Results	Correlation
Injured (*n* *=* 158)	42.03 ± 7.35	t(1019) = 3.030, *p =* 0.003	*r* *=* −0.093, (*p =* 0.003)
	(30–60)		
Non-injured (*n* *=* 863)	44.0 ± 7.56		
	(30–68)		
Illness (*n* *=* 296)	41.88 ± 7.48	t(1019) = 4.969, *p* *<* 0.001	*r* *=* −0.157, (*p* *<* 0.001)
	(30–65)		
No Illness (*n* *=* 725)	44.44 ± 7.47		
	(30–68)		
Injured/Illness (*n* *=* 390)	42.07 ± 7.38	t(1019) = 5.457, *p* *<* 0.001	*r* *=* −0.170, (*p* *<* 0.001)
	(30–65)		
Not injured/No Illness (*n* *=* 631)	44.69 ± 7.50		
	(30–68)		

## Abbreviations

The following abbreviation is used in this manuscript:

VJ: Vertical Jump
